# Single-cell RNA-Seq reveals transcriptional heterogeneity and immune subtypes associated with disease activity in human myasthenia gravis

**DOI:** 10.1038/s41421-021-00314-w

**Published:** 2021-09-14

**Authors:** Wanlin Jin, Qi Yang, Yuyao Peng, Chengkai Yan, Yi Li, Zhaohui Luo, Bo Xiao, Liqun Xu, Huan Yang

**Affiliations:** 1 Department of Neurology, Xiangya Hospital, Central South University, Changsha, Hunan China; 2 Department of Neurosurgery, Xiangya Hospital, Central South University, Changsha, Hunan China

**Keywords:** Autoimmunity, Transcriptomics

## Abstract

Myasthenia gravis (MG) is a rare autoimmune disease. Although the impact of immune cell disorder in MG has been extensively studied, little is known about the transcriptomes of individual cells. Here, we assessed the transcriptional profiles of 39,243 cells by single-cell sequencing and identified 13 major cell clusters, along with 39 subgroups of cells derived from patients with new-onset myasthenia gravis and healthy controls. We found that B cells, CD4^+^ T cells, and monocytes exhibited more heterogeneity in MG patients. CD4^+^ T cells were expanded in MG patients. We reclustered B cells and CD4^+^ T cells, and predict their essential regulators. Further analyses demonstrated that B cells in MG exhibited higher transcriptional activity towards plasma cell differentiation, CD4^+^ T cell subsets were unbalanced, and inflammatory pathways of monocytes were highly activated. Notably, we discovered a disease-relevant subgroup, CD180^−^ B cells. Increased CD180^−^ B cells in MG are indicative of a high IgG composition and were associated with disease activity and the anti-AChR antibody. Together, our data further the understanding of the cellular heterogeneity involved in the pathogenesis of MG and provide large cell-type-specific markers for subsequent research.

## Introduction

Myasthenia gravis (MG) is a rare autoimmune disease characterized by skeletal muscle weakness caused by disrupted neurotransmission at the neuromuscular junction, with a prevalence of 150–250 cases per 1 million^1^. B and T cell hyperactivity and the autoantibodies secreted by B cells mediate the autoimmune phenotype by responding to muscle neuronal nicotinic receptors^2^. In 80%-85% of MG patients, the pathogenic antibodies are anti-AChR^3^. CD4^+^ T cells, also known as T helper cells, are essential for helping antigen-experienced B cells produce these pathogenic high-affinity antibodies. The innate immune system also plays a significant role in these processes. Dendritic cells (DCs) can function as antigen-presenting cells and induce autoimmunity by promoting the expansion and differentiation of autoreactive T cells^4^. Autoantibodies produced by B cells can activate myeloid cells and form the proinflammatory milieu, which in turn promotes the disorder of adaptive auto-reactive T or B cells^5^.

Although major cell types involved in the pathogenic processes of MG are known, key cellular subsets, their transcriptomes characteristics, and the interactions through which they promote MG have remained largely unclear. Studies of the peripheral blood after sorting specific cell subtypes by flow cytometry in bulk also failed to capture the natural transcriptome signatures. Additionally, approximately 10% of MG patients are treatment refractory^6^, highlighting the need to better understand specific disease-associated pathogenic events. Furthermore, anti-AChR antibodies are markers for diagnosis and disease classification of MG patients but not for disease severity^7^. Therefore, additional markers are needed for indicating disease severity.

Here, we applied single-cell RNA sequencing (scRNA-seq) to visualize a high-resolution immune landscape of MG patients and compared them to healthy controls (HCs). We first identified 13 major cell groups and assessed the primary changes of these major types of cells. We next analyzed B cells and CD4^+^ T cells with more granularity due to their important roles in the adaptive immune process and the shift between MG patients and HCs. We characterized their cellular network to better understand their cellular identity and the process of their differentiation.

In summary, our analysis provides insights into the major immune cells in MG and HCs, the possible altered transcriptional differentiation trajectories, the higher connection to plasma cells in MG patients, and cellular cross-talk with potential relevance to pathogenic mechanisms. Moreover, our analysis defines a subset of B cells known as CD180^−^ B cells that clinically coincide with anti-AChR antibody and disease severity. We also showed that immunosuppressive therapy restored CD180^−^ B cells frequency. Finally, we investigated the relationship of major changed cell types and risk genes of MG patients, highlighting the increasing expression of HLA-DRA, HLA-DQA1, HLA-DQB1, and HLA-DPB1 in B cells of Chinese early-onset MG patients. Altogether, these analyses help characterize the cellular pathological mechanism by investigating cell differentiation and cellular interactions, identifying large cell type-based markers and pathways for understanding the pathogenic events that occur in MG to identify new effective therapeutics.

## Results

### Single-cell survey of major changes in transcriptional profiles between MG patients and healthy controls

**Fig. 1:**
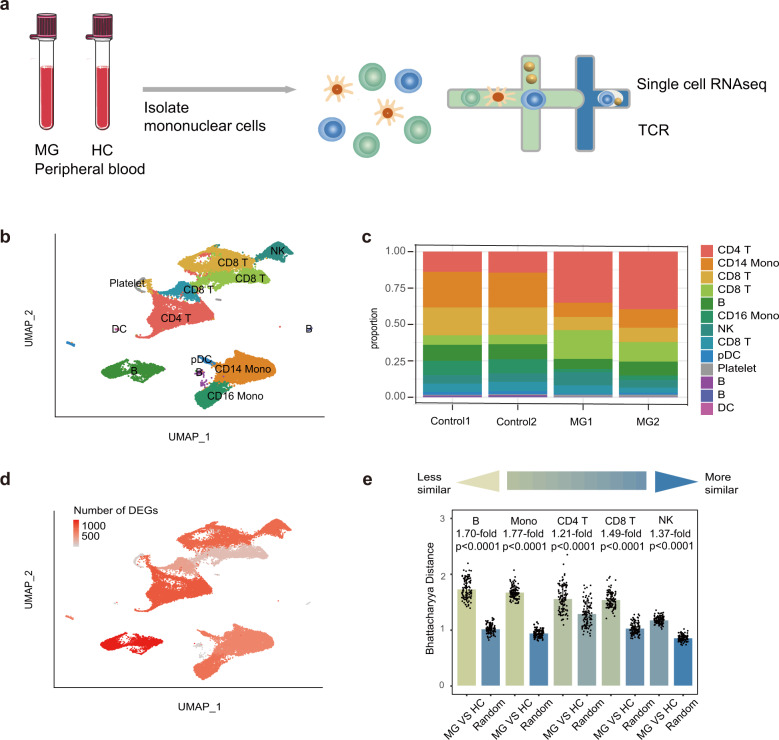
Assessment of major changes in transcriptional profiles between MG patients and healthy controls. **a** Overview of the scRNA-seq experiment: PBMCs were isolated from two healthy controls and two myasthenia gravis patients. Overall, ~39,243 cells were included in the functional analysis. **b** Uniform manifold approximation and projection (UMAP) representation of scRNA-seq data showing the seven main cell types: CD8^+^ T cells, CD4^+^ T cells, B cells, CD14^+^ monocytes, FCGR3A^+^ monocytes, NK cells, and DCs. **c** Cluster abundance across all samples. **d** Number of DEGs between MG and HC cells within each cluster projected onto the UMAP. DEG: |log fold change| > 0.5; *P-*value < 0.05 was calculated using DESeq2. **e** Quantification of differences between major immune cells between MG patients and HCs. Each dot represents a sub-sample of 500 cells from the principal component analysis space for MG patients and HCs. For the random groups, we sampled 500 cells of sample type. The height of the bar represents the mean values of the subsamples. All comparisons were significant due to 100 replicates of testing, while the mean fold change varied from 1.77-fold (monocytes) to 1.21-fold (CD4^+^ T cells). *P* values are from a Wilcoxon rank-sum test comparing the MG vs HC groups to the random selection for each type of major immune cell.

To characterize the immune changes in MG patients, we performed scRNA-seq and generated 39,243 high-quality single-cell transcriptomes of PBMCs from 2 early-onset MG (EOMG) patients and 2 HCs using the 10× Genomics platform (Fig. 1a, Supplementary Table S2). MG patients had not undergone immunotherapy treatment (Supplementary Table S1).

We first partitioned the single-cell profiles into 13 clusters composed of major cell subtypes, including CD8^+^ T cells, CD4^+^ T cells, B cells, CD14^+^ monocytes, FCGR3A^+^ monocytes, NK cells, and DCs (Fig. 1b, Supplementary Figs. S1, 2) using an unsupervised method. Cell types were identified by known unique signature genes, *CD3E* (T cells), *MS4A1* and *CD79A* (B cells), *CD14* and *FCGR3A* (monocytes), *LILRA4*, and *FCER1A* (DCs), and *NKG7* (NK cells) (Supplementary Fig. S1). A small population of platelets was also present after isolating PBMC preparations, which were excluded from further analysis.

We next assessed alterations in transcriptional profiles between MG patients and HCs in two ways. We calculated differentially expressed genes (DEGs) in each cluster (see Materials and Methods) and then projected the numbers of DEGs on UAMP. The DEGs revealed broad transcriptional changes in immune cells, with the most prominent in B cells and CD4^+^ T cells (Fig. 1d). In addition, we measured the distance between the major types of cells using Bhattacharyya distance^8^. DCs were not included due to the limited number of cells available. This result revealed large differences in B cells and monocytes (Fig. 1e) between MG patients and HCs, while CD4^+^ T cells, NK cells, and CD8^+^ T cells were more similar in this analysis. Actually, B cells exhibited the highest difference with 1.7-fold changes, followed by CD14^+^ monocytes with 1.67-fold changes (data not shown). Cell proportion analysis revealed that CD4^+^ cells were significantly expanded in MG patients compared to HCs (Fig. 1c). Overall, we identified the major immune cells and characterized broad changes of transcriptional profiles and cell proportions in MG patients compared to HCs, revealing prominent changes in B cells, CD4^+^ T cells, and monocytes.

### B cell clustering and subgroup analysis

**Fig. 2:**
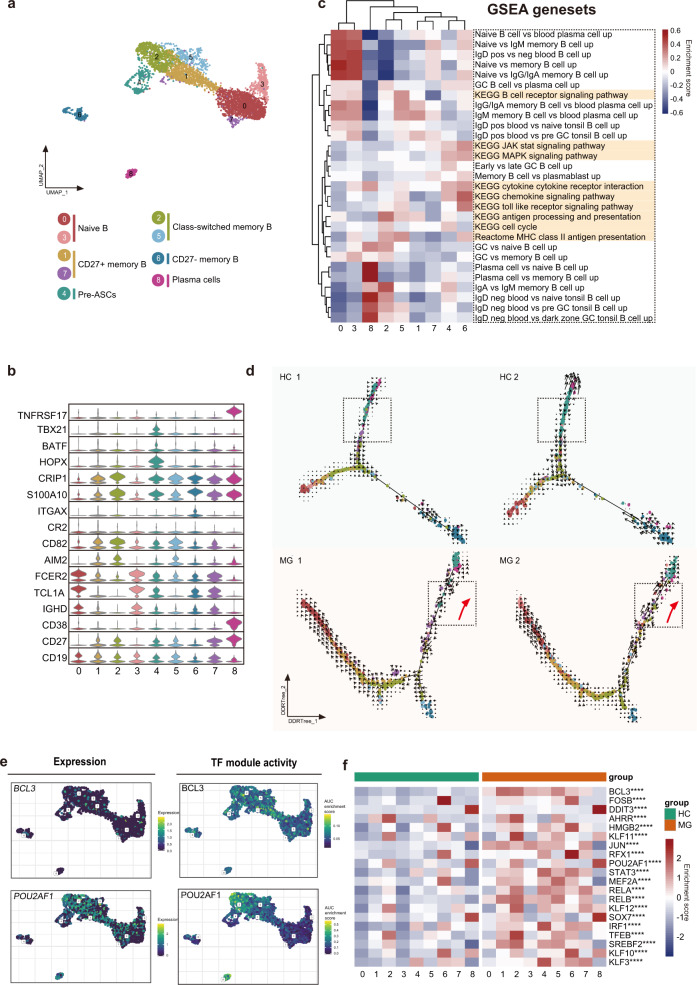
Assessment of changes in B cells in transcriptional profiles between MG patients and healthy controls. **a** UMAP plot displaying 4133 B cells from two MG patients and two HCs separated into 9 subtypes. **b** Violin plots showing key gene markers across B cell subsets. **c** Gene set enrichment revealed differences in pathway activities and cell identity of naive, memory, switched B cells, and plasma B cells. **d** B cells were sorted using the DDRTree algorithm and projected onto the different cell states using the color in **a**. Transcriptional activity was estimated by measuring the ratio between unspliced and spliced mRNAs. The length of the arrow represents the transcriptional activity. **e** UMAP plots showing the expression of *BCL3* and *POU2AF1* genes in B cells (top) and the AUC of the estimated regulon activity of the corresponding TFs, predicting the degree of expression regulation of their target genes (bottom). **f** Heatmap of the AUC scores of expression regulation by transcription factors (regulon activity), as estimated using SCENIC, followed by comparisons between two groups using the *t*-test. The twenty transcription factors with the highest upregulated expression in MG are shown.

We next bioinformatically separated and reclustered B cells. Clustering of B cells revealed 9 distinct clusters (Fig. 2a). Combined with reported marker genes^9,10^, we identified naïve B cells (clusters 0 and 3), class-switched memory B cells (clusters 2 and 5), CD27^+^ memory B cells (clusters 1 and 7), CD27^−^ memory B cells (cluster 6), pre-ASCs (cluster 4), and plasma cells (cluster 8) (Fig. 2b). We next assessed function by gene set enrichment analysis (Fig. 2c), which further supported the cluster annotation. Specifically, GSVA analysis revealed that cluster 6 was associated with cytokines, chemokines, toll-like receptor signaling pathway, and JAK-STAT and MAPK signaling. Cluster 5 was associated with B cell receptor signaling and major histocompatibility complex (MHC) class II antigen presentation. Cluster 4 was enriched in cell cycle set and intrinsic signaling, such as the JAK-STAT and MAPK pathways, implying that they were highly activated (Fig. 2c). This result was also consistent with the GO analysis indicating that clusters 4 and 8 were highly activated. KEGG analysis also showed that clusters 4 and 8 shared similar pathways (Supplementary Fig. S4). Furthermore, cluster 4 highly expressed *HOPX*, a marker of PrePB^10^. Thus, we infer that cluster 4 potentially represents a transient state before plasma cell transformation. Class-switched B cells (cluster 2) had undergone antibody class switching and were indicators of B cell activation by antigen stimulation, coincident with their gene enrichment in antigen processing and presentation process in GSVA analysis.

In addition, we comprehensively inferred transcription factor (TF) regulatory networks by SCENIC analysis, highlighted differentiation-associated TFs, and predicted additional markers. As expected, plasma B cells exhibited large transcription factor differences from their B cell predecessors, and the differentiation to plasma B cells is a process that loses B cell identity^11^. SPI1(PU.1), *ETS1*, *PAX5*, *SPIB*, and *BACH2* are transcription factors that represent B cell identities^11^ and loss of *PAX5* implies the ASC differentiation^12^. As expected, they are expressed in nonplasma cells. *STAT3*, which functions in IL-21 stimulated B cell differentiation^13^, are also not expressed on plasma B cells. Plasma cells have high regulon activity of *IRF4*, *Prdm1*, and *XBP1*, as expected. Additional increased TF regulon signatures included *CHD2*, *IRF7*, *ZBTB18*, *MYBL2*, *DDIT3*, and *ATF6B* for plasma cells. Plasma B cells and class-switched memory B cells have high regulon activity of *POU2AF1*, which is required for GC formation and thus has unique roles in T cell responses^14^ and generating ASCs^15^. This implies class-switched memory B cells are an important stage before the terminal ASCs to produce high-affinity antibodies. Pre-ASCs have regulon activity of *TBX21*, which positively regulates isotype switching to IgG isotypes. Additional possible markers for B cell subsets are provided in the supplement (Supplementary Fig. S6).

### Extensive B cell heterogeneity and altered differentiation trajectories

Class-switched B cells were expanded in MG. GSVA analyses revealed that they participate in antigen processing and presentation, supporting that class-switched B cells are highly activated, and their function is increased in MG patients (Fig. 2c, Supplementary Fig. S3).

**Fig. 3:**
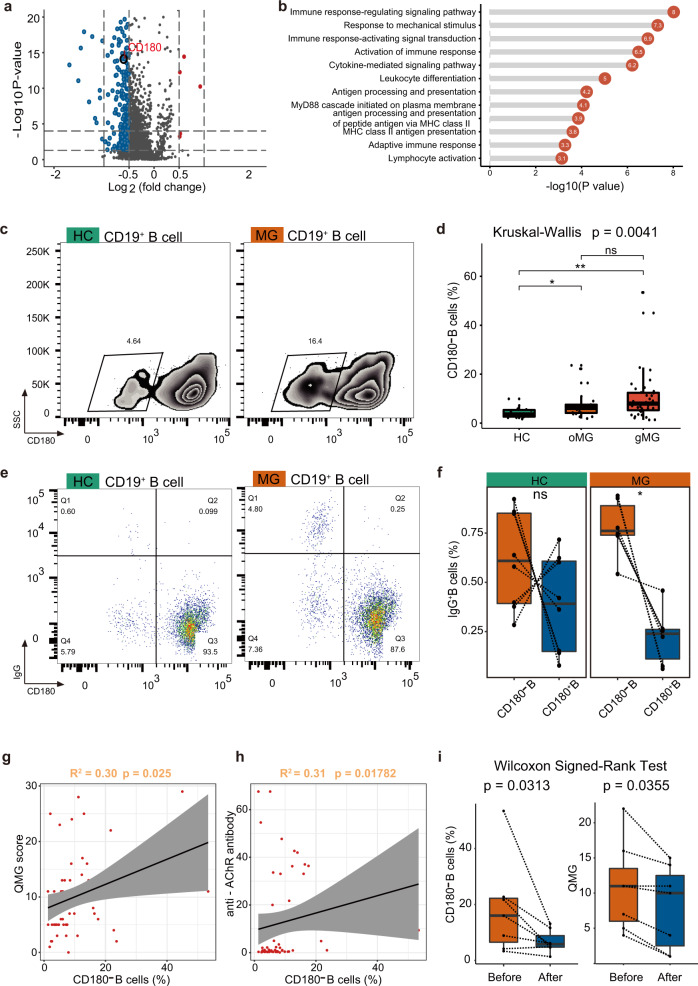
A unique B cell subpopulation associated with AChR antibody and disease activity in MG patients. **a** Volcano plot showing DEGs between MG patients and HCs. DEG: |log fold change| > 0.5; *P* value < 0.05 was calculated using DESeq2. **b** Enrichment analysis of DEGs from B cells between MG patients and HCs (selected among upregulated pathways in MG patients, *P* value < 0.05). **c**, **d** CD180^−^ B cells are increased in MG patients. **e**, **f** CD180^−^ B cells are the major IgG secreting cells in MG patients and HCs. CD180^−^ IgG cells are significantly higher than CD180^+^ IgG cells in MG patients. **g** Circulating CD180^−^ B cells are associated with disease activity. **h** Circulating CD180^−^ B cells are associated with titers of anti-AChR antibodies. **i** Immunotherapy decreases CD180^−^ B cells and is associated with improved disease activity.

To characterize transcriptomic changes in B cells between MG patients and HCs, as well as their biological significance, DEGs were calculated (Fig. 3a) and a detailed analysis of the DEGs was performed by the functional annotation of Gene Ontology (GO) (Fig. 3b). Pathways related to antigen processing and presentation and antigen processing and presentation of peptide antigen via MHC class II were upregulated, driven by *HLA-DPA1*, *HLA-DQB1*, and *HLA-DRB5*. *HLA-DPA1* and *HLA-DQB1* genes, which are also reported predisposing risk genes in Chinese MG patients^16^. Also upregulated pathways were involved in leukocyte differentiation and activation, with elevated *ZFP36L2*, *IRF1*, *JUN*, *JUNB*, *RELB*, *CD83*, *HLA- ZFP36L2*, *IRF1*, *JUN*, *JUNB*, *RELB*, *CD83*, *HLA-DPA1*, *EZR*, *DUSP1*, and *CXCR4* genes. The CXC-chemokine receptor 4 (*CXCR4*), which is important for the recruitment of ASCs^17^, was increased in MG patients, implying the increasing homing process of tissues. Overall, antigen presentation, immune response signaling, and differentiation pathways were activated, with cytokine participation contributing to deficits in peripheral immune tolerance.

To reveal the programming processes with alterations in B cells, we used the Monocle 2 method to construct the differentiation trajectory of B cells from each sample (Fig. 2d). In HCs, trajectory analysis revealed a gradual transition from naïve B cells (cluster 0, 3) to the fate of plasma B cells (cluster 8) or memory B cells (cluster 6), with most cells undergoing differentiation to plasma B cells. In MG patients, while the differentiation pathways were similar, cluster 4 was on average closer to the terminal cluster 8 and seemed to have an aberrant developmental process. Cluster 4 highly expressed *BATF*, without which B cells fail to induce Aicda and differentiate into plasma B cells^18^. Cluster 4 also highly expressed *TBX21*, and a previous study demonstrated that T-bet is expressed by memory pre-ASCs^19^. RNA velocity further indicated that higher numbers of unspliced RNAs were present in the naïve cells of MG patients (Fig. 2d). Moreover, we observed increased activity towards plasma B cells in MG patients. The above analysis revealed the more highly activated state of naïve B cells and the enhanced propensity for differentiation towards a plasma B cell phenotype in MG patients.

Next, to assess the TFs underlying differences in B cells between MG and HCs, we applied SCENIC analysis (Fig. 2e, f). Among the 20 most elevated TFs, BCL3, and POU2AF1 are associated with differentiation. Eμ-BCL-3 transgenic mice with BCL-3 overexpression led to a hyperactivated state of B cells^20^. Furthermore, the top enriched BCL3 also serves as a unique factor to control NF-kB activity in the nucleus^21^, suggesting a role for immune activation and inflammatory regulation. Indeed, the activities of nuclear factor (NF)-κB subunits (RELB) were also highly upregulated in B cells from MG patients. These results also support the prominent feature of MG patients wherein B cells are highly activated and exhibit aberrant differentiation states.

### Correlations of B cell subtype-specific signatures with disease activity and autoantibodies

Notably, DEGs revealed decreased expression of the *CD180* gene in MG patients compared to HCs (Fig. 3a) (log2Fc = −0.61, adj. *P* = 2.92E−13). Flow cytometry analysis further supported that the frequencies of CD180 negative B cells were significantly increased in both ocular and generalized MG patients (*P* = 0.004). The gating strategy is shown in Supplementary Fig. S5. The frequencies of CD180 negative B cells were not different between ocular and generalized MG patients (Fig. 3c, d), suggesting that the increased CD180 negative B cells might be a common pathological mechanism of MG.

Evaluating disease severity by the Quantitative Myasthenia Gravis (QMG) score^22,23^, the CD180 negative B cells correlated with disease severity (*R*^2^ = 0.30, *P* = 0.025) in MG patients (Fig. 3g). The increased CD180 negative B cells were also associated with anti-AChR autoantibodies (*R*^2^ = 0.31, *P* = 0.018) (Fig. 3h). As anti-AChR antibodies primarily belong to the IgG1 and IgG3 subclass^24^, we examined the IgG secreting B cells by intracellular flow cytometry. The results showed that though CD180 negative B cells were the major IgG secreting B cells, CD180 positive B cells still stained slightly positive for intracellular IgG and still existed in both MG patients and HCs. However, CD180 negative B cells from MG patients demonstrated significantly increased IgG secreting ability compared to CD180 positive B cells (*n* = 6, *P* = 0.031), while no significance was observed in HCs (*n* = 8, *P* = 0.313) (Fig. 3e, f).

Assessing treatment efficacy by QMG score^25^, after immunosuppressive therapy, CD180 negative B cells decreased (*P* = 0.031), and patients exhibited clinical improvement along with decreased QMG scores (*P* = 0.036) (Fig. 3i). In summary, we identified a cell group that may have an important role in anti-AChR autoantibody secretion and indicated disease activity.

### T cell and NK cell clustering and subgroup analysis

**Fig. 4:**
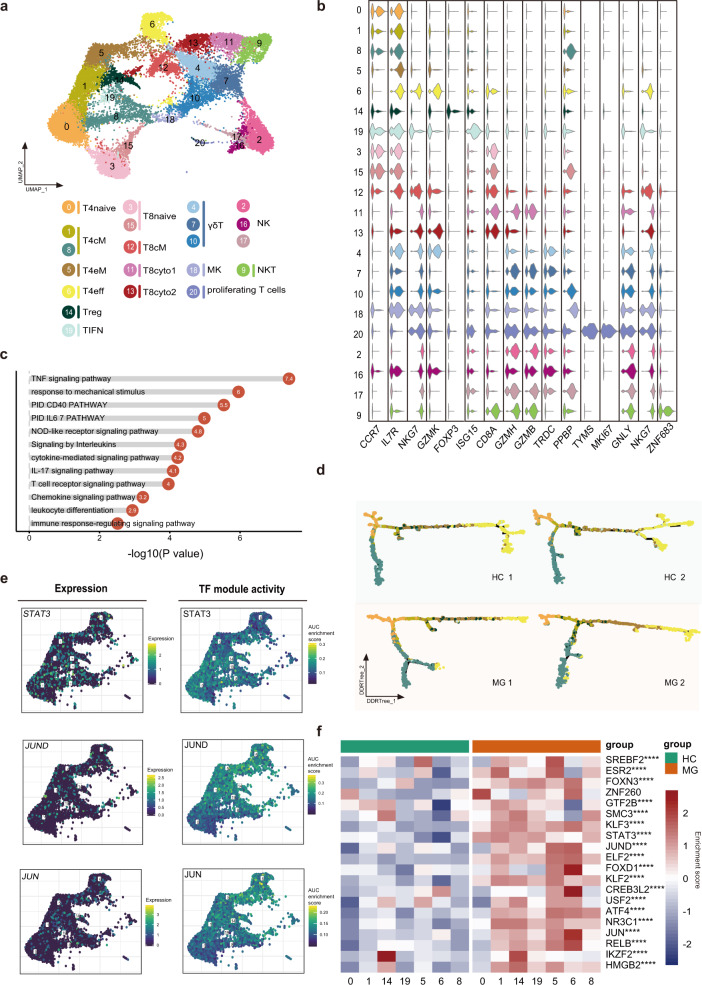
Assessment of changes in T cells in transcriptional profiles between MG patients and healthy controls. **a** UMAP plot displaying T cells and NK cells from two MG patients and two HCs separated into 21 subtypes. **b** Violin plots showing key gene markers across T cells and NK cells. **c** Enrichment analysis of DEGs in CD4^+^ T cells between MG patients and HCs (selected among upregulated pathways in MG patients, *P* value < 0.05). **d** CD4^+^ T cells were sorted using the DDRTree algorithm and projected onto the different cell states using the color in **a**. **e** UMAP plots showing expression of the *STAT3*, *JUND*, and *JUN* genes in CD4^+^ T cells (top) and the AUC of the estimated regulon activity of the corresponding TFs, predicting the degree of expression regulation of their target genes (bottom). **f** Heatmap of the AUC scores of expression regulation by transcription factors (regulon activity) as estimated using SCENIC, followed by comparisons between two groups using *t*-test. The top 20 transcription factors with the highest upregulated expression in MG are shown.

We subgrouped T cells and NK cells into 21 subsets and named them according to reported marker genes. Seven CD4^+^ T cell clusters (0, 1, 5, 6, 8, 14, 19), 5 CD8^+^ T cell clusters (3, 11, 12, 13, 15), 3 γδ T cell clusters (4, 7, 10), 1 MK cluster (18), 1 NKT cluster (9), proliferating T cell cluster (20) and 3 NK cell clusters (2, 16, 17) were identified (Fig. 4a).

In the T cell compartment, the combination of the GSVA analysis results and reported marker genes identified a CD4^+^ T cell subpopulation of naïve CD4+ cells expressing *CCR7* (T4naive, cluster 0), central memory CD4^+^ T cells (T4cM, cluster 1), effector memory CD4^+^ T cells (T4eM, cluster 5) with low expression of C*CR7*, effector CD4^+^ cells (T4eff, cluster 6) expressing *NKG7* and *GZMK*, regulatory (T4reg, cluster 14) and interferon-activated cells (TIFN, cluster 19) highly expressing *ISG15*. In addition, cluster 8 highly expressing *CCR7* belonged to naïve CD4^+^ T cells or central memory CD4^+^ T cells. CD8^+^ T cells included naïve CD8^+^ cells (T8naive, clusters 3 and 15) expressing *CCR7*, central memory CD8^+^ T cells (T8cM, cluster 12) expressing *IL7R*, cytotoxic CD8 cells (T8cyto1, clusters 11) expressing *GZMH* and *GZMB*, cytotoxic CD8 cells (T8cyto2, cluster 13) expressing *GZMK* and γδ T cells (clusters 4, 7, 10) expression high levels of *TRDC*. Additional populations included a small group of megakaryocyte-like cells (MK, cluster 18) expressing *PPBP* and cluster 20, which represented proliferating cells with high expression of *TYMS* and *MKI67* (Fig. 4b). MG patients exhibited higher proportions of T4cM and T4eM.

We next used SCENIC to identify TF regulatory networks. Naïve CD4^+^ T cells highly expressed *UQCRB*, *TCF7*, *CHD2*, and *MYC*. Interferon-activated cells highly expressed *IRF1*, *STAT1*, and *STAT3*. Previous studies have shown that IFN-γ activating *STAT1* is important for Th1 differentiation^26^, and *STAT3* is involved in Th17 differentiation and maintenance^27^. *IRF1* can regulate the differentiation and expansion of Th1 and Th17^28^. Combining the high expression of *IL17A* in clusters 1, 5, and 19, TIFN may represent the Th1 and Th17 states. *HMGB1*, which was previously demonstrated to directly enhance immune inhibitory functions of Tregs^29^, was highly expressed in Tregs. Interestingly, T4eM and T4eff shared similar expression patterns for many TFs, suggesting that the T4eff may be derived from T4eM. Our data characterize the regulators of the CD4^+^ T cell subgroup, which helps to further investigate the modulation of CD4^+^ T cell activation and plasticity.

### CD4^+^ T cell heterogeneity

Since CD4^+^ T cells were significantly expanded in MG patients and given their essential roles in stimulating B cells to produce high-affinity antibodies, we determined the transcriptional changes between MG patients and HCs. DEGs were calculated and followed by enrichment analysis using Metascape (Fig. 4c). The results showed that TNF signaling, NOD-like receptor signaling, IL-17 signaling, T cell receptor signaling, and CD40 pathways were highly enriched in MG patients, indicating that Th17 cells and T helper type 40 (TH40) cells were expanded and exhibited greater antigenic stimulation of peripheral CD4^+^ T cells in patients with MG compared to HCs. Murine studies highlighted the importance of Th17 cells for their contribution to the loss of B-cell tolerance^30^.

To investigate the relationships between the different states of CD4^+^ T cells, differentiation trajectory analysis (Fig. 4d) was performed and showed that naïve T cells and central memory T cells primarily aggregated on the pseudotime backbone. Cytotoxic CD4^+^ T cells and cluster 8 were primarily located in different directions. The direction to cluster 8 might represent the quiescent state, while the other represents steps to the terminal stages. Interestingly, effector CD4^+^ T cells exist in both ends of the two directions in myasthenia gravis, while they primarily exist in one direction after the effector memory CD4^+^ T cells or Treg stage and have more branches in HCs. Previous murine research demonstrated that disequilibrium of the CD4^+^ helper T-cell subsets promoted the development of EAMG^31^. Comparing the differentiation progression of CD4^+^ T cell subsets between MG and HCs, CD4^+^ T cell subsets except for TIFN showed extensive differences (Supplementary Fig. S7). Our result also indicated that CD4^+^ T cells from MG patients exhibit imbalance in CD4^+^ T cell subgroups from their transcriptomes.

We next explored transcription factors (TFs) in CD4^+^ T cells that might be involved in promoting autoimmunity (Fig. 4e, f). Among the 20 most highly elevated TFs, we observed that regulon activity of STAT3, JUND, and JUN, which are associated with differentiation, was elevated in MG patients. JUND reportedly regulates lymphocyte proliferation and Th cell cytokine expression^32^. Our results identify key transcription factors associated with changes in CD4^+^ T subgroups.

### Myeloid clustering

**Fig. 5:**
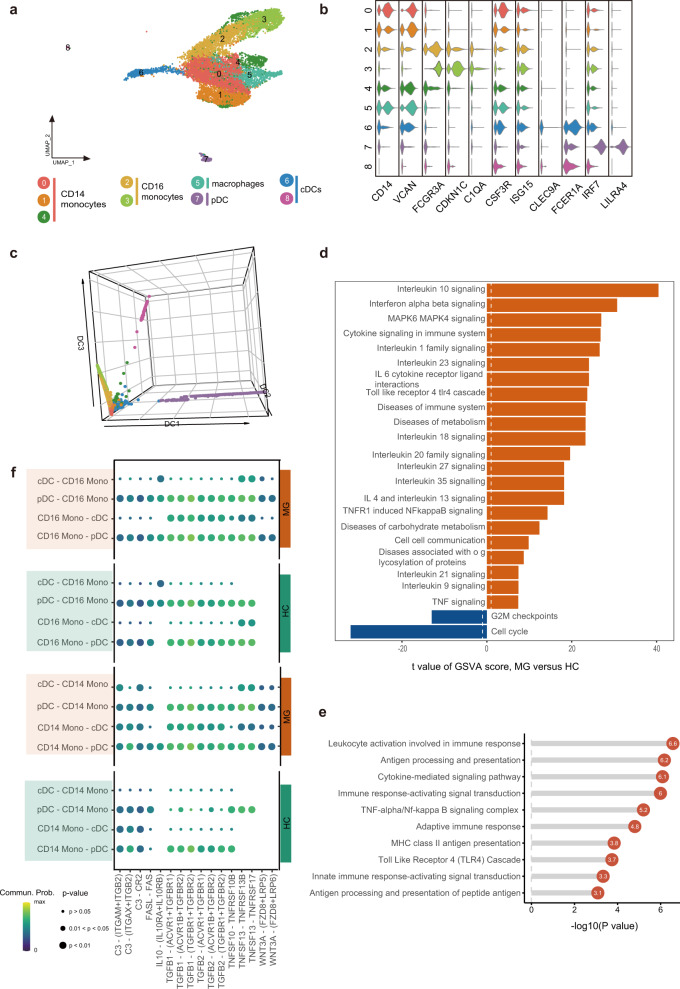
Assessment of changes in myeloid cells in transcriptional profiles between MG patients and healthy controls. **a** UMAP plot displaying myeloid cells from two MG patients and two HCs separated into 9 subtypes. **b** Violin plots showing key gene markers across myeloid cells. **c** 3D diffusion map displaying the developmental trajectory of myeloid cells. The result shows the possible activation paths of macrophages/cDCs. Cells are colored by their derived clusters. pDCs, plasmacytoid dendritic cells; cDCs, conventional DCs. **d** Enrichment analysis of DEGs in dendritic cells between MG patients and HCs (selected among upregulated pathways in MG patients, *P* value < 0.05). **e** GSVA analysis of CD14^+^ monocytes between MG patients and HCs. **f** Dot plot of selected significant paracrine receptor-ligand interactions between CD14^+^/CD16^+^ monocytes and cDCs discovered using *Cellchat*. Commun.Prob., communication probability.

11,507 myeloid cells were detected in MG and HCs. Myeloid cells were then sub-grouped into 9 clusters (Fig. 5a, b), including three CD14^+^ monocytes (clusters 0, 1, and 4) and two CD16^+^ monocytes (clusters 2 and 3). Three myeloid subsets were defined as DCs, including two conventional DCs (cDCs) highly expressing *CLEC9A* or *FCER1A* (clusters 6 and 8) and one plasmacytoid dendritic cells (pDC) highly expressing *IRF7* and *LILRA4* (cluster 7). We designated one myeloid subset as macrophages according to high expression of *CSF3R* and *ISG15* (cluster 5).

We constructed a diffusion map to depict the developmental trajectory of myeloid cells (Fig. 5c). CD14^+^ monocytes are the root cell group. The diffusion map of monocytes/macrophages/DCs revealed three developmental paths, possibly corresponding to the macrophage, cDC, and pDC phenotypes, respectively.

### Myeloid heterogeneity analysis

CD14^+^ monocytes, which can further differentiate into CD16^+^ monocytes, macrophages, or DCs, exhibited large differences between MG patients and HCs using Bhattacharyya distance. DEG analysis of CD14^+^ monocytes showed that MG patients express high levels of the inflammatory markers *S100A4*, *S100A8*, *S100A9*, *S100A10*, and *S100A12*. We found most distinguishing pathway changes between MG patients compared to HCs were inflammatory-relevant pathways, including MAPK family signaling, TNF signaling, TLR4, interferon, and interleukin signaling (Fig. 5d). Furthermore, diseases of metabolism were also enriched in MG patients, such as disorders of glycosylation and carbohydrate metabolism.

As expected, we found that the genes with increased expression in DCs in MG patients were enriched in antigen processing and presentation, cytokine-mediated signaling pathway, and TNF-alpha/nuclear factor k-light-chain-enhancer of activated B cells (Nf-kappa B) signaling (Fig. 5e). Nf-kappa B signaling activation controls the expression of critical co-stimulatory molecules, such as CD80, CD86, and MHC class II, along with pro-inflammatory cytokines, such as TNF-alpha. Our result emphasized the importance of Nf-kappa B signaling in DCs in myasthenia gravis.

MG alters crosstalk between monocyte and cDC subpopulations. CDCs can derive from CD16^+^ and CD16^−^ monocytes and CD16^+^ monocytes can derive from CD14^+^ monocytes. Previous work reported that an estimated 25% of the circulating inflammatory monocytes will differentiate into migrating DCs^33^, thus we explored cDC, CD14^+^ monocyte, and CD16^+^ monocyte receptor–ligand pairing in both MG patients and HCs, which may represent the differentiation of myeloid cells. We found altered receptor–ligand pairing between CD14^+^ or CD16^+^ monocytes and cDCs other than pDCs (Fig. 5f), suggesting that the differentiation of circulating monocytes into cDCs might be modulated through TNF signaling and TGF-β signaling and the cDCs derived from monocytes might be with different phenotypes and functional profiles in MG patients.

### Global comparison analysis of communications among immune cells

Excluding cell-intrinsic information, scRNA-seq can also indicate putative cell-extrinsic interactions by integrating ligand and receptor information. We used *CellChat* to investigate the putative interactions between the major types of immune cells in MG versus HCs^34^.

**Fig. 6:**
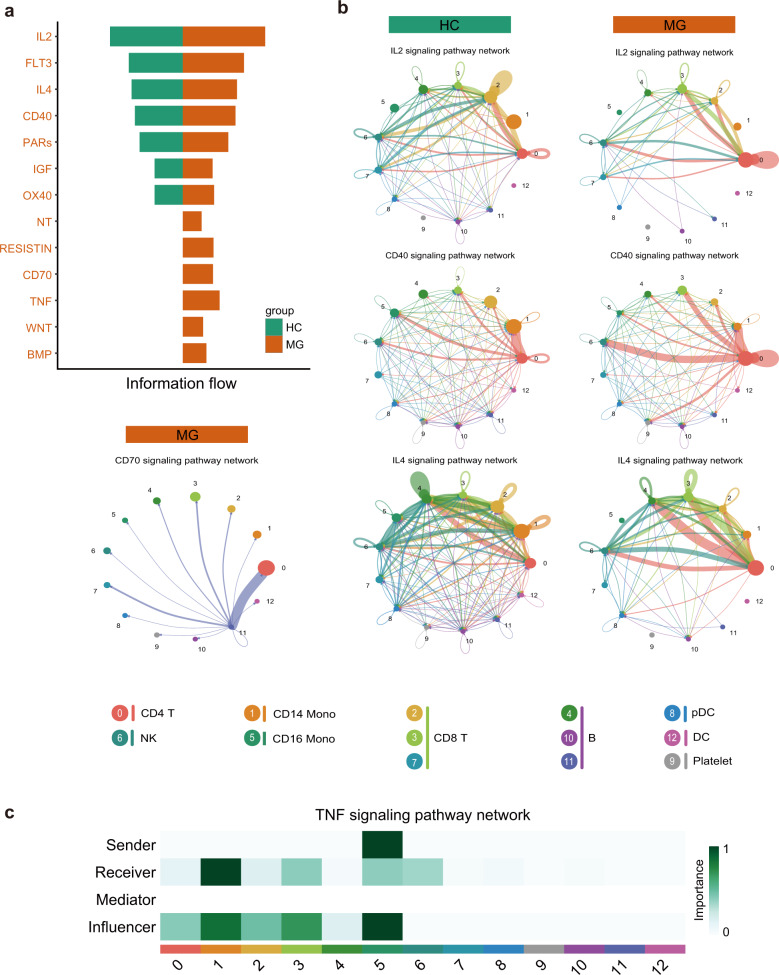
Evaluation of expression of genes for receptors and ligands, and cell-cell communication. **a** The significant signaling pathways were ranked based on their differences in overall information flow within the inferred networks between HCs and myasthenia gravis patients. We show the more enriched signaling pathways colored in orange in myasthenia gravis. **b** Circos plot showing the inferred intercellular communication network across major cell types using the color of **b** for HCs and MG patients. **c** Heatmap showing the relative importance of each cell group based on the computed four network centrality measures of the TNF signaling pathway. Information flow refers to the overall communication probability.

We identified 13 significant signaling pathways among the 13-cell group by *CellChat*. The top signaling pathways that are more enriched in MG patients are colored orange. Circos plots were used for visualization for the specific interactions among the 13-cell group. The results showed increasing activities of pathways, including IL2, IL4, CD40, CD70, BMP, RESISTIN, TNF, WNT, and NT, in MG patients (Fig. 6a). In addition, CD4^+^ T cells had increasing autocrine activity for soluble IL2 and had a massive expansion of interactions with other cell types through the CD40 signaling pathway in MG patients. Another interesting observation is that IL4 pathways from CD4^+^ T cells to B cells were enriched. Our result proved the T cell also increasingly helping to B cells in peripheral blood except for germinal centre in MG patients. Since the loss of IL-4 was associated with loss of IgG1^35^, the increasing IL-4 might lead to the increasing IgG1 in MG patients. Further analysis showed that the CD70 signaling pathway was most enriched from B cells to CD4^+^ T cells in MG patients (Fig. 6b). CD16^+^ monocytes are the prominent influencer controlling TNF signaling, and the TNF signaling pathway was most enriched from CD16^+^ monocytes to CD14^+^ monocytes (primarily TNF-TNFRSF1B) (Fig. 6c). These findings are consistent with the known critical roles played by myeloid cells in initiating inflammation. Notably, *CellChat* predicted that WNT and NT signaling pathways are from a source of both lymphoid cells and myeloid cells, including CD4^+^ T, CD8^+^ T, and CD14^+^ monocytes, and they have similar targets. This reveals that WNT signaling in MG is complex and redundant with multiple ligand sources. CD4^+^ T cells are the predominant source and mediator for WNT signaling, suggesting their potential roles as a gatekeeper of WNT signaling in MG (Supplementary Fig. S8).

### Expression of MG-relevant genes and biological pathways

Previous GWAS studies have linked genetic variants to MG; however, the cellar and biological processes of the risk genes are little known due to large variants. We collected large GWAS studies of MG patients and identified variants in MHC class II locus, protein tyrosine phosphatase nonreceptor type 22 (*PTPN2*2), TNFAIP3 interacting protein 1 (*TNIP1*)^36^, cytotoxic T-lymphocyte–associated protein 4 gene (*CTLA4*)^37^, combining with the predisposing gene of EOMG including *HLA-DRA*, *HLA-DR3*, *HLA-B8*, *HLA-DPB1*, *HLA-DQB1*, *HLA-DQA1*, *CD86*, *AKAP12*, *VAV1*, *TNFSF13B (*B-cell activating factor, *BAFF*), and *TNF*^37–40^. We also included the α-subunit of the AChR encoding gene *CHRNA1*^41,42^, IgG receptor genes (*FCGR2A*, *FCGR3A*, and *FCGR3B*), and cytokines and cytokine receptors genes (*TNFB*, *TNF*^43^*, IL1B*^44^, *IL1A*^45^, *IL10*^46^, *IFNG*, *IL17A*, and *IL17F*^47^).

**Fig. 7:**
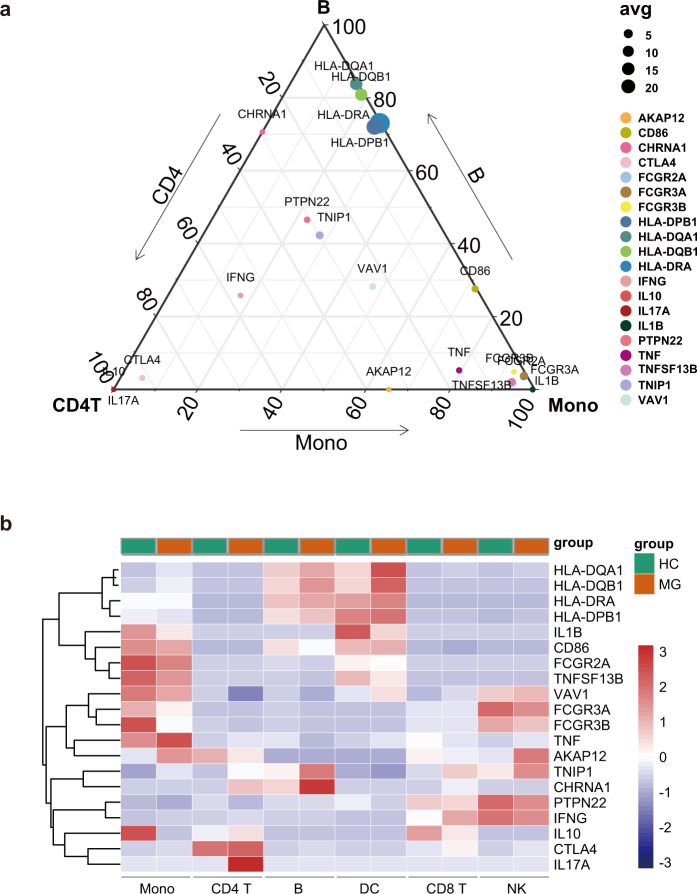
Cell-type specificity of MG-associated genes using microfluidics scRNA-seq. **a** Expression of 20 genes associated with myasthenia gravis by cell type in MG patients. HLA genes have the highest expression in B cells and DCs. **b** Ternary plot of the 20 risk genes of two MG patients, comparing CD4^+^ T cells (left), B cells (top), and monocytes (right). The color of each node represents different genes, where the node size represents the average expression of the three cell types.

Here, we applied transcriptomic atlas to relate the patterns of MG genetic risk with patterns of cell-specific expression (Fig. 7a, b). Some risk genes were not available in our data, and 20 genes were ultimately analyzed. While the human leukocyte antigen (HLA) locus remains the most strongly associated risk factor for MG and is associated with autoantibody expression^41^, relevant genes, including *HLA-DPB1*, *HLA-DQB1*, *HLA-DQA1*, and *HLA-DRA*, are expressed predominantly in DCs and B cells. The *CHRNA1* gene is expressed mainly in B cells, with a small amount of *CHRNA1* expression in CD4^+^ T cells. A previous study also reported that *CHRNA1* and *HLA-DQA1* are associated with autoantibody titers^48^, further emphasizing the importance of B cells. Genes with functions that regulate T cell activation^49^ are expressed most highly in CD4^+^ T cells (e.g., *CTLA4*, *IL17A, IL10*). Another T cell activation regulatory gene, *PTPN22*, is highly expressed in CD8^+^ T cells. *IL1B*, involved in the modulation of autoimmune inflammation^50^, is primarily expressed in monocytes and DCs.

Several genes associated with immune or inflammatory signaling of autoimmune disease, including *VAV1*^38^ and *TNIP1*^51^, are expressed in multiple cell types. *TNF*, *TNFSF13B*, and *FCGR2A* are highly expressed in monocytes, which as previously reported are associated with the autoimmune state of monocytes^52^. *AKAP12*, associated with pathway activation, was primarily expressed in monocytes and NK cells. *IFNG*, *FCGR3A*, and *FCGR3B* were primarily expressed in NK cells and monocytes. *CD86* was highly expressed in monocytes, DCs, and B cells.

Combined with the DEG analysis, the significantly altered expression of MG risk genes might lead to impaired functions. For example, *HLA-DRA* was upregulated in B cells, DCs, and CD8^+^ T cells. *HLA-DQA1* was upregulated in B cells, DCs, CD8^+^ T cells, and monocytes. *HLA-DQB1* was upregulated in B cells, DCs, and monocytes. *HLA-DPB1* was upregulated in B cells, CD8^+^ T cells, monocytes, and NK cells. Overall, the HLA class II genes are differentially expressed in B cells of myasthenia gravis, suggesting their function of pathogenic antigen presentation. Further studies are needed to elucidate the functional changes of these specific cells in MG.

## Discussion

In this study, we aimed to understand the cellular transcriptional changes in MG patients. We are the first to create a high-resolution atlas and to systematically discuss the cellular heterogeneity and the impaired peripheral tolerance from MG patients. We performed scRNA-seq of 39,243 cells and then annotated their cell type identity, followed by DEG and pathway analysis. At this resolution, we first identified 13 cell clusters and subsequently reclustered them into 39 subsets. We found general differences in monocytes, CD4^+^ T cells and B cells, while NK cells and CD8^+^ T cells were more similar between MG and HCs. Based on the 39 clusters, we also compared the differentiation shift and observed aberrant B cell and CD4^+^ T cell differentiation by *monocle2* and RNA velocity. We found large cell type-based DEGs, which might be referenced for investigating the pathological roles of immune-cell subsets. The scenic analysis provides clues for identifying new candidate TFs involved in B cell and T cell dysfunction. Cell communication analysis also provides potential drug targets for treating the disease.

Our approach to transcriptional profiling of the peripheral blood in myasthenia gravis patients is unique because it did not involve presorting. This unbiased method uses the full transcriptome and might provide a more detailed and comprehensive overview. We next noted several key observations. We found that B cell differentiation was highly connected with plasma cells. DEGs and metascape analysis revealed increasing antigen processing and presentation and MHC II molecular expression, including *HLA-DRA*, *HLA-DQA1*, *HLA-DQB1*, and *HLA-DPB1*, which are also MG-associated risk genes.

Notably, we found that CD180 is decreased in MG patients. We further verified this with an independent cohort by flow cytometry and found a large expansion of CD180^−^ B cells in MG patients, which was correlated with anti-AChR antibodies and disease activity. A similar phenomenon with the expansion of peripheral CD180^−^ B was also observed in SLE^53^. Our results showed that CD180^-^ B cells were the IgG-secreting B cells both in MG and HCs. We further showed that CD180 negative B cells exhibited increased IgG-secreting ability. Previous studies reported that CD180 knockout mice have one-tenth the serum concentrations of IgG3 than WT mice^54^, further indicating the importance of the *CD180* gene for IgG secretion. In summary, CD180 expression is altered in B cells and might have an important role in autoantibody secretion. CD180^−^ B cells may serve as indicators for the disease activity of MG. In addition, we revealed some TFs may regulate the observed aberrant differentiation and activity of B cells in MG patients, including BCL3, POU2AF1, JUN, RELB, and STAT3. Few of these TFs and their function on B cells were researched in MG, but some TFs were reported in other immune diseases and most of the TFs are associated with antibody-secreting. *Bcl-3*-deficient Mice have a decreased B cell population, impaired formation of GCs, and severe defects in the production of antigen-specific antibodies^55^. *POU2AF1*(*Bob1*)-sufficient mice lead to abrogated germinal center B cell formation, anti-CII antibody production, and Collagen-induced arthritis development^56^. Consistent with data from animal studies, Expression of *POU2AF1* mRNA was elevated in B from synovial fluid of rheumatoid arthritis patients and was strongly correlated with *CD21L*, a molecular marker of GCs^56^. In humans with *RelB* deficiency, patients suffer from severe immunodeficiency with shortage-specific antibodies due to the halted B cell development and the absent CD27^+^ memory B cells^57^. In B6.MRL/lpr mice, *STAT3* deficiency led to the defect of plasma cell differentiation and decreased autoantibody production^58^.

For T cells, we also observed expansion and altered differentiation of CD4^+^ T cells. The highly enriched IL-17 signaling pathway and CD40 pathway in MG patients suggested the expansion of Th17 and CD40^+^ CD4^+^ T cells. Similar to a previous study that showed CD40 can be expressed on CD4^+^ T cells. Furthermore, CD40^+^ CD4^+^ cells are reported to be highly pathogenic and promote broad TCR repertoire expansion in autoimmune type 1 diabetes^59,60^, which partly explains the increased TCR signaling. Cell communication results revealed increased CD70 signaling between plasma B cells and CD4^+^ T cells. CD27 is expressed on the majority of T cells and is upregulated with T cell activation, and its ligand CD70 is transiently expressed on activated B cells. CD27 ligation can promote effector cell formation. This might suggest activated B cells also promote CD4^+^ T subgroup alterations. Moreover, we revealed some TFs may regulate CD4^+^ T-cell polarization in MG, such as STAT3, JUND, and JUN. CD4^*STAT3*^^−/−^ mice are resistant to experimental autoimmune encephalomyelitis and the inability of pathogenic Th17 and Th1 cells^61^. *JUND* also promoted Th17 cells polarization, although *JUND* is not major role compared with *JUNB*^62^.

Although previous large studies of pathology in MG focused on adaptive immunity, we observed that monocytes also showed large shifts in MG patients. Apart from enriched antigen processing and presentation activity, DCs also exhibited activated TNF signaling. Cell communication showed increased TNF signaling from CD16^+^/CD14^+^ monocytes to cDCs. Monocytes might have functions in mediating the TNF signaling of DCs. TNF signaling plays an important role in inflammatory and autoimmune diseases and participates in biological processes, including cell proliferation, apoptosis, and differentiation. Their altered function might be the cause of the chronic inflammatory reaction. We propose that monocytes also play an essential role in the pathological process of MG.

There are also limitations to our study. First, due to the limitation of RNA capture efficiency, there were undetected genes. For example, some risk genes that are lowly expressed were not identified. Second, our scRNA-seq dataset included only female early-onset MG patients to avoid gender bias, which may not share the same pathological transcriptional changes with other disease subtypes or male patients. Third, clustering based on the expressed mRNA transcriptome may not always be coincidence with clustering by protein surface markers.

In summary, we present a comprehensive single-cell transcriptome atlas of MG. Our study helps better understand the mechanism of MG for basic research, provides indicators of disease activity for clinicians, and provides markers for drug developers.

## Materials and methods

### Processing of patient samples

Sixty-two MG patients and, seventeen age- and sex-matched HCs were recruited at the Neurology Department of Xiangya Hospital between June 2018 and February 2019. The first cohort including HCs (*n* = 2) and MG (*n* = 2), was used for the 10× genomics scRNA-seq. The second cohort, including HCs (*n* = 15) and MG (*n* = 60), was used for flow cytometry analysis (Supplementary Table S1). Seven MG patients in the second cohort were followed up after 3 months of immunosuppressant therapy. Participants were excluded based on the following criteria: (1) an ethnic origin other than Han; (2) a history of oral glucocorticoid or immunosuppressant intake; (3) a history of IVIG or plasma therapy; (4) a history of malignant tumor; (5) pregnancy. Diagnosis of MG was based on clinical symptoms, neostigmine test, repetitive nerve electrical stimulation, and serum autoantibody results. Autoantibody results, including AChR and MuSK antibody, were obtained from the DAAN Clinical Laboratory Central (Guangzhou, China). Serum anti-AChR levels greater than 0.45 nmol/L and anti-MuSK levels greater than 0.5 nmol/L were considered positive results. QMG score was used to evaluate disease severity when patients have enrolled and the therapeutic effect after immunotherapy treatment^22^. All patients and HCs signed informed consent forms. This study was approved by the Ethics Committee of Xiangya Hospital.

### Generation and sequencing of single-cell libraries

Peripheral blood mononuclear cells (PBMCs) were isolated from whole blood using Ficoll-Paque (TBD, Tianjin, China) according to the manufacturer’s instructions. Cell viability was assessed by flow cytometry (cell viability > 95%). ScRNA-seq libraries were prepared per the Chromium Single Cell 5' library preparation kit user guide (10× Genomics). 90 μL cellular suspension, 40 μL barcoded Single Cell 5' Gel Beads, and 270 μL Partitioning Oil were loaded onto a Chromium Chip A to generate single-cell gel bead-in-emulsions (GEMs). GEM-reverse transcriptions (GEM-RTs) were performed in a Veriti 96-well thermal cycler (Thermo Fisher Scientific). After RT, cDNAs were amplified and cleaned using the SPRIselect Reagent Kit. Indexed sequencing libraries were constructed using the Chromium Single Cell 5' Library Construction Kit (10× Genomics) according to the user guide. Barcoded sequencing libraries were quantified by quantitative PCR on an ABI StepOnePlus Real-Time PCR System (Life Technologies). Pair end single-cell RNA-Seq libraries were sequenced using NovaSeq 6000 (Illumina) with a read length of 150 bp paired-end reads.

### ScRNA-seq bioinformatics analysis

#### Quality control metrics and filtering

For each sample, we processed 10× genomics raw data using the Cell Ranger Single-Cell Software Suite (v 3.1.0) with default parameters. Reads were aligned to the prebuilt human reference genome from the 10× Genomics website (GRCh38 V3.0.0). Then, we mapped the unique molecular identifiers (UMIs) to genes and the barcodes to cells. We further analyzed these metrics using the *R* (v3.6.3) package *Seurat* (v 3.1.2). Only genes expressed in at least three cells and cells with a minimum of 200 genes were kept. Low-quality cells meeting one of the following thresholds were further excluded: 1) the number of expressed genes was lower than 500 or larger than 3500; 2) more than 7% of UMIs were mapped to mitochondrial or ribosomal genes. After filtering, we detected 18928 genes in a total of 39,243 cells, as shown in Supplementary Table S2.

#### Dimensional reduction and visualization

After quality control, variable genes for each four samples were identified using the FindVariableGenes function in *Seurat*. Then, single-cell transcriptomes data were log-normalized and scaled for further analysis. FindIntegrationAnchors and IntegrateData functions were used to produce a batch-corrected expression matrix^63^. Principal component analysis (PCA) for dimension reduction was performed with the *Seurat* function, and 20 PCs were kept for downstream analysis based on the Jackstraw method. Clustering was further performed with the *Seurat* FindClusters function, and the Uniform manifold approximation and projection (UMAP) was used for visualization. Identities of clusters of cells were manually annotated using known marker genes in published articles with the help of the R package *SingleR* (v 1.0.1). Sub-clustering of major immune cell clusters was performed in the same workflow.

#### Differential expression analysis

Cluster-specific marker genes for transcriptional subpopulations were identified by the FindAllMarkers *Seurat* function with Wilcoxon rank-sum test. For specific cluster comparisons between MG patients and HCs, we used the *DESeq2* package (v1.26.0) to detect differentially expressed genes (DEGs)^64^. Significant DEGs were filtered by |log fold change| > 0.5 and *P* value < 0.05. Gene ontology and gene-set enrichment analysis from DEGs were performed using Metascape (www.metascape.org)^65^.

#### Gene functional annotation

To compare the functional profiles of different clusters, we used the *clusterProfiler* package (v 3.14.3)^66^ for Gene Ontology (GO) and Kyoto Encyclopedia of Genes and Genomes (KEGG) pathway analysis with significant DEGs. Gene set variation analysis (GSVA) was performed using the *GSVA* package^67^ (v 1.32.0). To assess the gene set activity among clusters, we first determined the mean expression of gene sets of each cluster and assessed the log fold-change between clusters. The annotation gene sets were downloaded from C2 (curated gene sets) and C7 (immunologic gene sets) of the molecular signature database (MSigDB, version 7.0). We used *Limma* package (v 3.42.0)^68^ to contrast the activity score and then compared pathway activities between MG and HCs.

### Quantifying differences between major immune cells in MG versus HCs

After identifying the major immune cells, we also evaluated the differences between lineages derived from MG versus HCs by comparing cell distributions. Bhattacharyya distance was used to measure the distances, and lineages with over 500 cells in each sample of MG and HCs were calculated as reported by Cillo et al.^8^. More specifically, for the comparison of any given two clusters, we first sampled 500 cells from each individual cluster randomly 100 times. Then, two clusters were mixed up, and 500 cells were randomly sampled twice without replacement, and inter-cluster Bhattacharyya distances were calculated between sampling of individual clusters and normalized with a sampling of mixed clusters.

### Regulatory network inference

A single-cell regulatory network for 9 B cell groups and 7 CD4^+^ T cells was performed with single-cell regulatory network inference and clustering (*scenic*)^69^. Specifically, *GRNBoost2* (https://github.com/tmoerman/arboreto) in *pySCENIC* was used to identify gene regulatory networks, and *RcisTarget* (v 1.6.0) was then used to identify regulatory motifs and to predict candidate target genes. The cell-regulon activity was calculated by *AUCell* (v 1.8.0). Differences in the AUC between the MG and HC groups were identified by *t*-test.

### Pseudotime-trajectory analysis

We used Monocle2 (v2.14.0)^70^ to construct the pseudotime-trajectories for B cells and CD4^+^ T cells. The positive significant marker genes identified by FindAllMarkers Seurat function were used to sort cells in a temporal differentiation order. Dimension reduction was performed using DDRTree from *Monocle2*.

A diffusion map for myeloid cells was generated using the *destiny* (v3.0.1) *R* package^71^.

### RNA velocity analysis

We used velocyto.py (v0.17, https://github.com/velocyto-team/velocyto.py) to quantify spliced and unspliced reads of each 10× bam file generated by cell range with default parameters. Expressed repetitive elements in the GRCh38 reference genome were masked, and the bed file for the masked interval was generated with the UCSC genome browser (https://genome.ucsc.edu/)^72^. Loom files were then processed with velocyto.R (v0.6 https://github.com/velocyto-team/velocyto.R). We subset B cells for further analysis with the grouping of 20 cells and used Monocle DDRTree embedding for the final RNA velocity plots, and cell to cell distance was calculated with the first 50 PCs. Parameters were set to default unless stated otherwise.

### Assessment of receptor/ligand interactions

We use CellChat (v0.0.2)^34^ to evaluated cell-cell interactions and significant pathways. To identify potential cell-cell interactions that were perturbed or induced in myasthenia gravis patients, we focused on differentially expressed ligands and receptors in CD8^+^ T cells, CD4^+^ T cells, B cells, CD14^+^ monocytes, FCGR3A^+^ monocytes, NK cells, and DCs. We also evaluated potentially altered interactions between monocytes, including CD14^+^ and CD16^+^ monocytes, and DCs, including cDCs and pDCs, based on the reclustered myeloid cells. Briefly, we followed the official workflow, and normalized data were loaded into *CellChat*. After creating *CellChat* objects, we used *CellChatDB.human* and set the *Secreted Signaling* pathways as the database. Then, default parameters were used to identify putative interaction pairs, and the result were displayed as circos plots.

### Flow cytometry

Flow cytometry was used to assess B cell subsets. PBMCs were isolated by standard Ficoll-Paque (TBD, Tianjin, China). Fresh PBMC from the donors were stained with the surface markers of anti-human antibodies, including BB515-labeled anti-CD19 (clone: HIB19, Biolegend), and PE-labeled anti-CD180 (clone: MHR73-11, Biolegend) antibodies, and standard procedures were applied. For intracellular staining, cells were fixed and permeabilized using BD Cytofix/Cytoperm (BD Biosciences) and then incubated with PEcy7-labeled IgG (clone: HP6017, Biolegend). Zombie (Biolegend) was used to exclude dead cells. Cells were subsequently blocked in 2% bovine serum albumin (BD Biosciences) supplemented with human FcX Blocker (Biolegend). 1 × 10^6^ fresh cells were used for every staining, and cells were incubated in staining buffer (0.1% BSA in PBS) for 30 min at 4 °C shielded from light. After staining, cells were washed in 1× PBS and acquired on a Cytek flow cytometer in an hour. Data were analyzed using FlowJo v10.

### Statistical analysis

Marker genes for transcriptional subpopulations were identified by FindAllMarkers Seurat function with Wilcoxon rank-sum test. Differences among multiple groups were evaluated by one-way analysis of variance (ANOVA) followed by Newman–Keul *post hoc* test. Wilcoxon signed-rank test was used for paired data. The paired Wilcox signed-rank test was used to compare differences between paired samples. Correlation analysis was performed using the nonparametric Spearman test. Statistical analysis and graph processing were performed using *R* (v3.6.3). Data were considered significant at *P < 0.05, **P < 0.01, and ***P < 0.001 unless stated otherwise.

## Supplementary information

Supplementary Information Information

## Data Availability

The accession numbers for the sequencing raw data in this paper are GSA (Genome Sequence Archive in BIG Data Center, Beijing Institute of Genomics, Chinese Academy of Sciences): HRA000997
