# Supplementary material for: Single-cell RNA-Seq reveals transcriptional heterogeneity and immune subtypes associated with disease activity in human myasthenia gravis
Source: Cell Discov. 2021 Sep 14;7:85. doi: 10.1038/s41421-021-00314-w (PMC8440681; doi:10.1038/s41421-021-00314-w)
Supplement: Supplementary file 1 — Supplementary Information Information [file 41421_2021_314_MOESM1_ESM.pdf]

Supplement

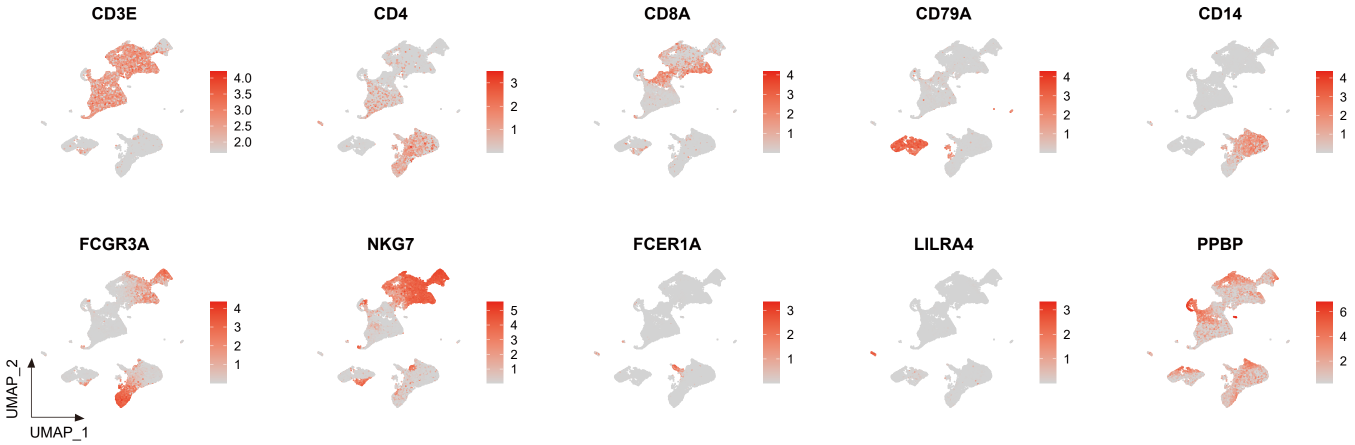

Fig. S1 UMAP projections of well-known marker genes

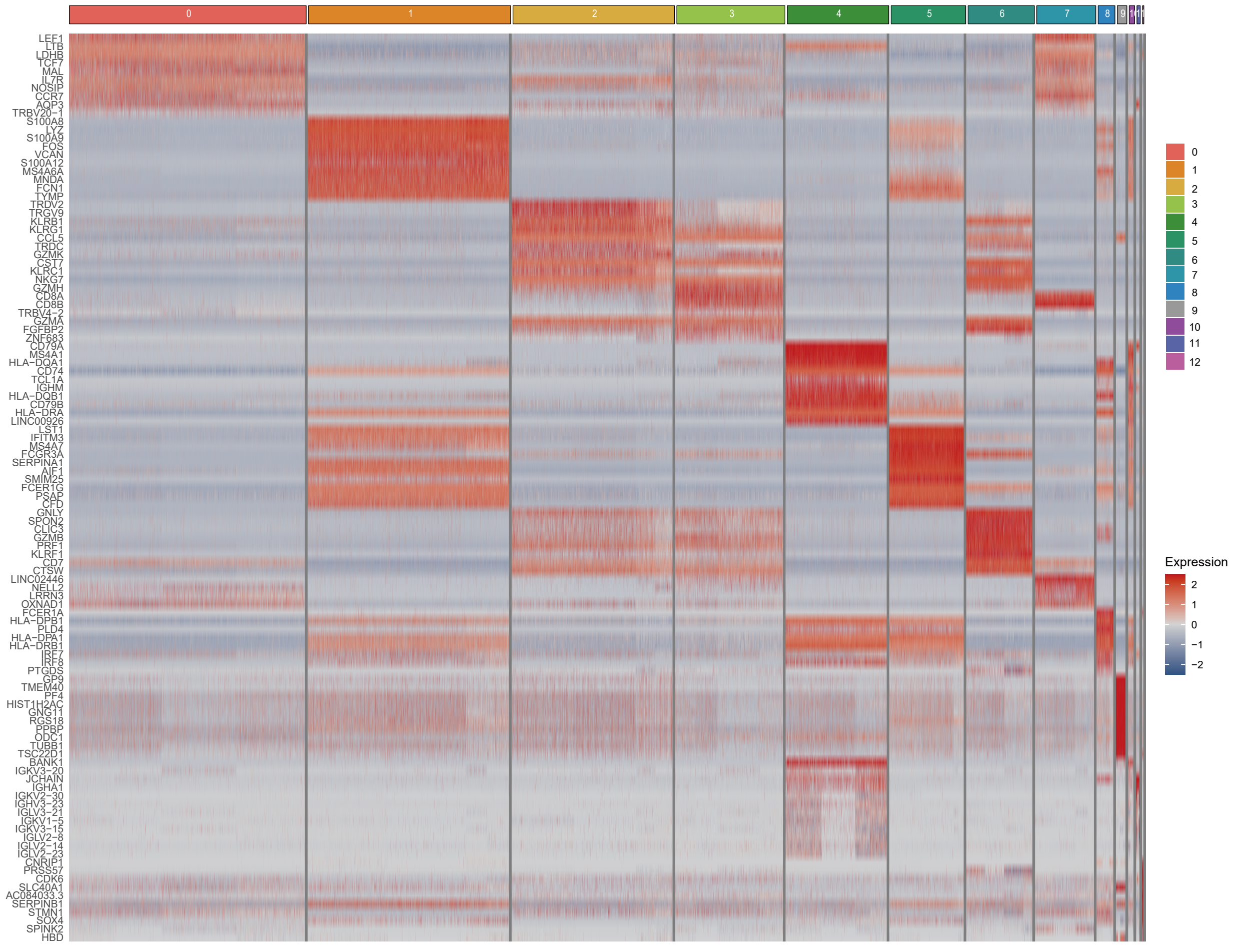

Fig. S2 A heatmap shows genes (rows) that are differentially expressed across 13 individual clusters (columns). Red: high expression; blue: low expression.

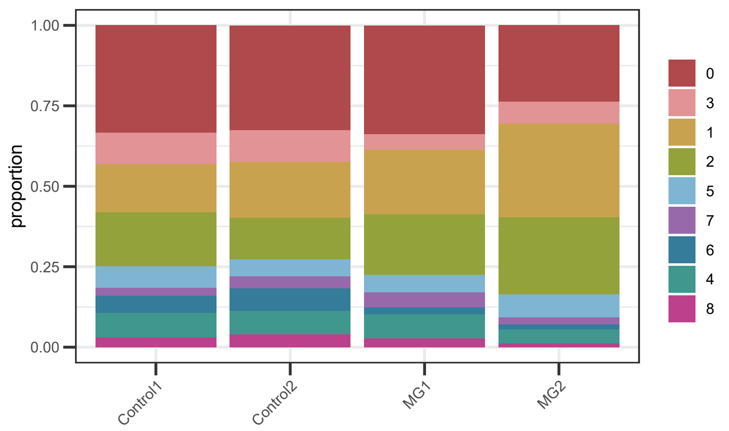

Fig.S3 Cluster abundances across all samples

a

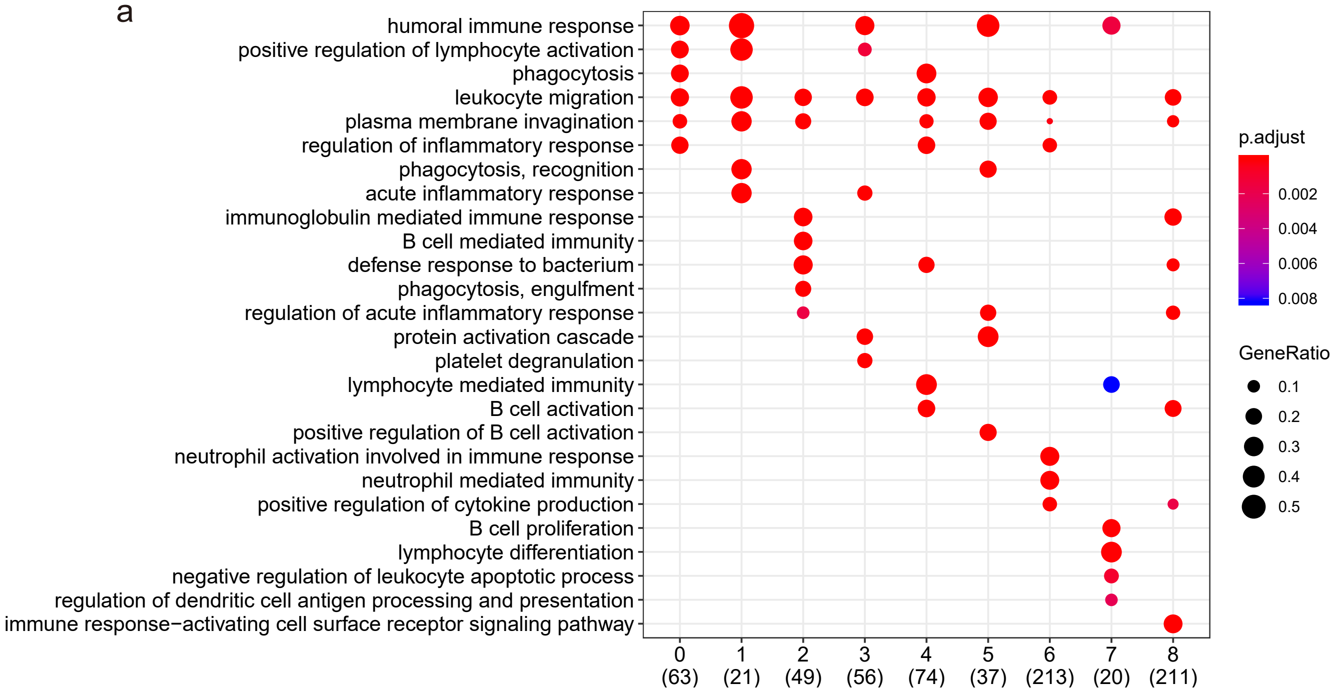

b

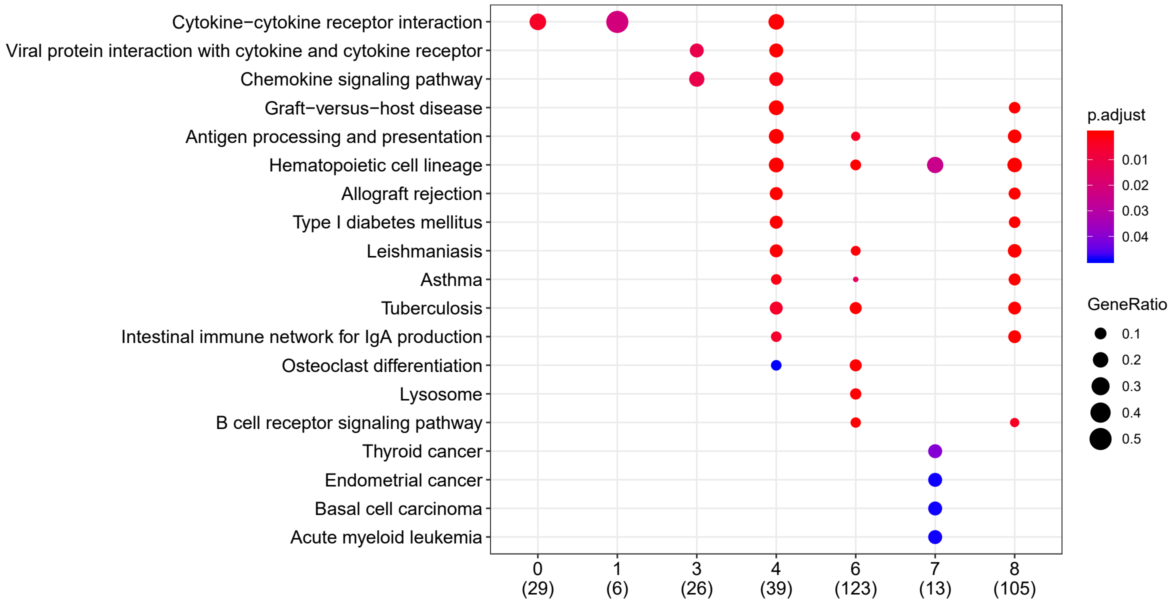

Fig. S4 GO analysis (a) and KEGG enrichment analysis (b) for B cell subsets. 0-8 represent cluster 0-8.

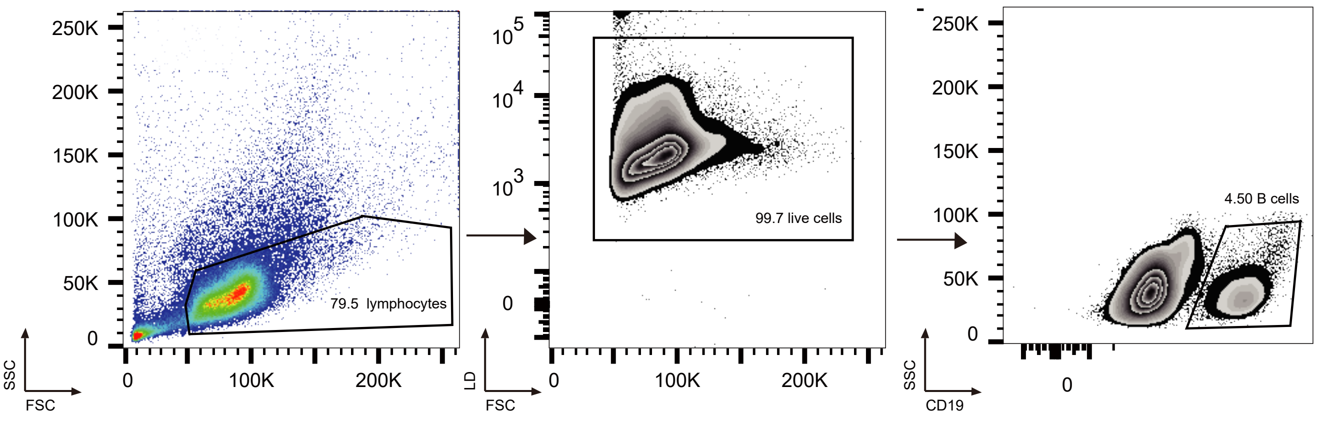

Fig. S5 Gating strategy for B cell flow cytometry

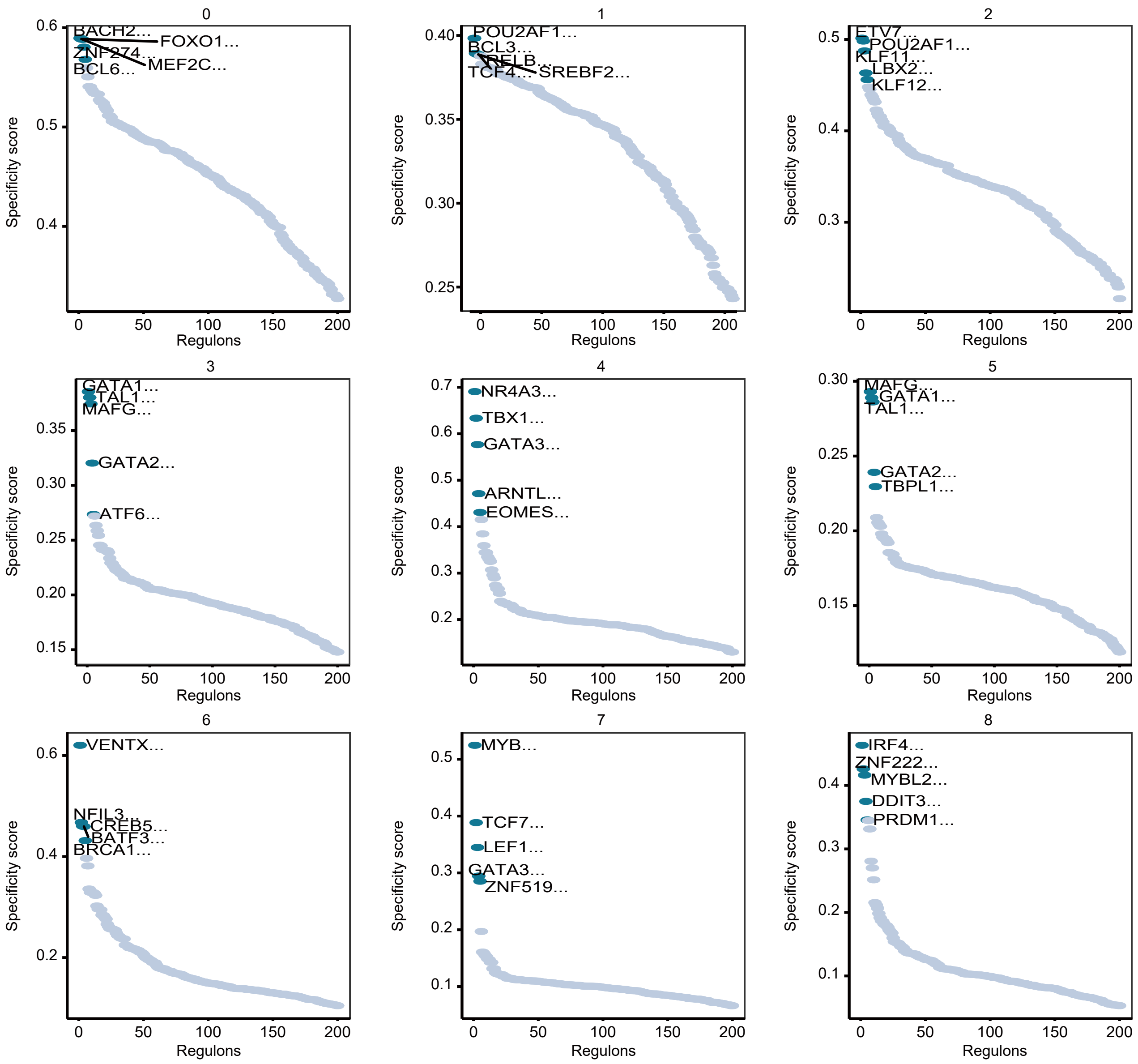

Fig. S6 Cell-Type-Specific Regulon Activity Analysis for B cell subsets. Rank for regulons in B cell based on regulon specificity score (RSS).Regulon specificity score (RSS) is calculated based on Jensen-Shannon Divergence.[1] 0-8 represent cluster 0-8.

1. Suo, S., Q. Zhu, A. Saadatpour, et al., "Revealing the Critical Regulators of Cell Identity in the Mouse Cell Atlas" Cell Rep, vol. 25, no. (6), pp. 1436-1445.e3,2018.

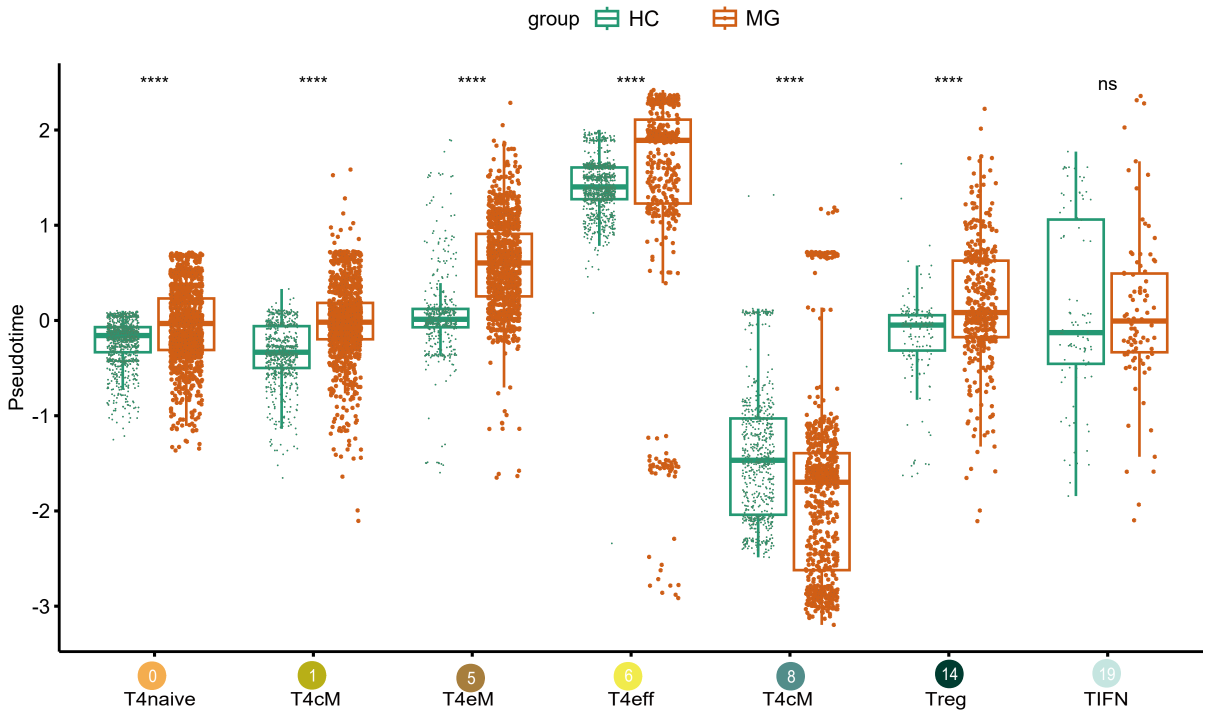

Fig. S7 Boxplot of pseudotime values of CD4⁺ T cell subsets between HCs and MG patients. * indicates a significant difference between HC and MG pseudotime value distribution (Wilcoxon rank sum test)

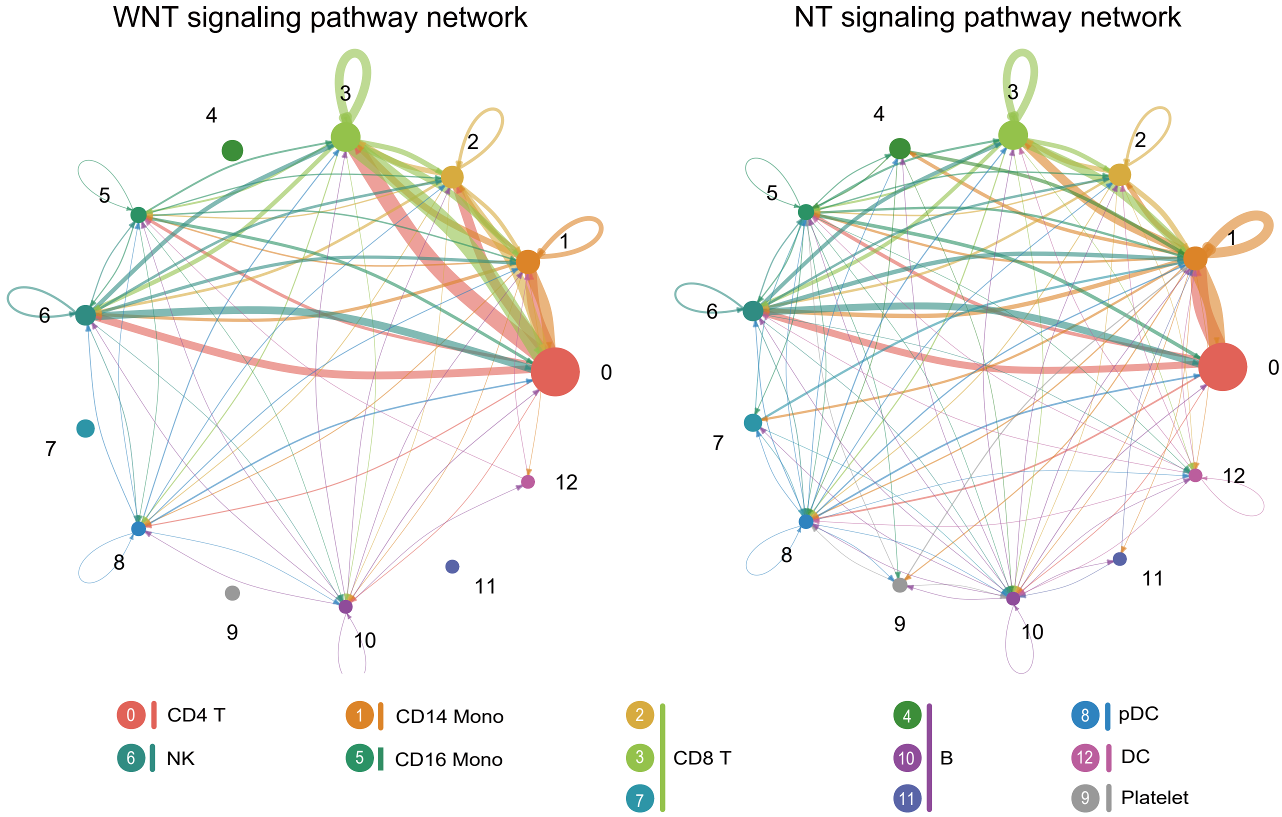

Fig. S8 Circos plot showing the inferred intercellular communication network of WNT/NT signaling pathway across major cell types in MG patients using the color of Fig. 1b.

Supplemental table 1

Demographic characteristics of study populations

Study cohorts	Subject	Case No.	Sex (M/F)	Age (range)	OMG	GMG	QMG score(mean± SD)	AChR-Ab positive	Thymoma
I	HC	2	0/2	37 (22-52)					
	MG	2	0/2	33 (18-48)	0	2	8±1.41	2	0
II	HC	15	7/8	24 (20-64)					
	MG[a]	60	26/34	39(14-81)	21	39	9.9 ± 7.0	41	3

a 7 MG patients from cohort II were follow-up 3 months after mmunosuppressants treatment

OMG, ocular myasthenia gravis; GMG, generalized myasthenia gravis; HC, healthy controls

AChR-Ab, nicotinic acetylcholine receptor antibody; MuSK-Ab, muscle specifc tyrosine kinase antibody

Supplemental table 2

Name	control1	control2	mg1	mg2
Estimated Number of Raw Cells	15066	15276	10004	10122
Mean Reads per Cell	18745	17934	31070	34872
Median Genes per Cell	1116	1148	1499	1203
Number of Reads	282408188	273958113	3.11E+08	3.53E+08
Valid Barcodes	94.00%	93.70%	92.70%	93.50%
Sequencing Saturation	73.20%	70.00%	78.10%	83.00%
Q30 Bases in Barcodes	94.60%	94.40%	94.50%	97.00%
Q30 Bases in RNA Read	91.80%	91.60%	91.80%	95.40%
Q30 Bases in RNA Read2	88.60%	88.40%	88.10%	93.80%
Q30 Bases in UMI	94.20%	94.00%	94.20%	96.50%
Reads Mapped to Genome	92.30%	91.50%	91.80%	94.10%
Reads Mapped Confidently to Genome	88.80%	87.70%	86.10%	88.80%
Reads Mapped Confidently to Intergenic Regions	6.00%	6.20%	6.80%	7.60%
Reads Mapped Confidently to Intronic Regions	8.00%	7.80%	6.50%	7.10%
Reads Mapped Confidently to Exonic Regions	77.30%	76.00%	75.00%	76.70%
Reads Mapped Confidently to Transcriptome	72.00%	70.90%	69.80%	71.70%
Reads Mapped Antisense to Gene	2.60%	2.50%	2.60%	2.30%
Fraction Reads in Cells	92.40%	91.90%	93.10%	91.10%
Total Genes Detected	20356	20438	19989	19855
Median UMI Counts per Cell	2755	2885	4000	3445
Cells after Filtering	12320	12369	8013	6541
